# Consensus based recommendations for the management of post-COVID long-term sequelae (Long COVID): a regional perspective

**DOI:** 10.3389/fmed.2025.1453167

**Published:** 2025-08-06

**Authors:** Majid AlShamrani, Fayssal Farahat, Abdullah Assiri, Sami AlHajjar, Ali Albarrak, Hashem AlHashemi, Mohammed AlZunitan, Meshal AlAqeel, Sami AlYami, Ziad A. Memish

**Affiliations:** ^1^Infection Prevention and Control Program, King Abdulaziz Medical City, King Abdullah International Medical Research Center, King Saud bin Abdulaziz University for Health Sciences, Ministry of National Guard Health Affairs, Riyadh, Saudi Arabia; ^2^Ministry of Health, Riyadh, Saudi Arabia; ^3^Pediatric Infectious Diseases, Department of Pediatrics, King Faisal Specialist Hospital and Research Center, College of Medicine, AlFaisal University, Riyadh, Saudi Arabia; ^4^Department of Internal Medicine and Infectious Diseases, Prince Sultan Military Medical City, Riyadh, Saudi Arabia; ^5^Department of Rehabilitation Medicine, King Abdulaziz Medical City, King Abdullah International Medical Research Center, King Saud bin Abdulaziz University for Health Sciences, Ministry of National Guard Health Affairs, Jeddah, Saudi Arabia; ^6^Section of Psychiatry, King Abdulaziz Medical City, King Abdullah International Medical Research Center, King Saud bin Abdulaziz University for Health Sciences, Ministry of National Guard Health Affairs, Riyadh, Saudi Arabia; ^7^Department of Respiratory Medicine, King Abdulaziz Medical City, King Abdullah International Medical Research Center, King Saud bin Abdulaziz University for Health Sciences, Ministry of National Guard Health Affairs, Riyadh, Saudi Arabia; ^8^King Salman Humanitarian Aid & Relief Center (KSrelief) and College of Medicine, Alfaisal University, Riyadh, Saudi Arabia; ^9^Hubert Department of Global Health, Emory University, Atlanta, GA, United States; ^10^Abu Dhabi University, Abu Dhabi, Saudi Arabia

**Keywords:** Long COVID, post-acute sequelae of COVID-19 (PASC), post COVID-19 condition (PCC), recommendations, management

## Abstract

**Background and objective:**

The coronavirus disease 2019 (COVID-19) pandemic marks the biggest public health emergency of the 21^st^ century. The long-term health issues linked to COVID-19, also known as Long COVID, pose a great challenge to patients and society. This article aims to highlight the current unmet clinical needs and present expert recommendations for region-specific assessment and management of Long COVID.

**Methods:**

A secondary desk research was performed focusing on global and regional guidelines for the assessment and management of Long COVID. The observations from the desk research form the foundation for the consensus statements. Additionally, an expert panel consisting of 10 consultant physicians from related specialties reviewed these guidelines and literature in advisory board meetings to identify unmet needs, bridge clinical gaps, and develop recommendations for the evaluation and management of Long COVID.

**Results:**

The expert recommendation statements were drafted based on overarching principles for the clinical assessment and management of Long COVID. The overarching principles used a targeted, multidisciplinary approach, and collaboration between multiple specialties of healthcare. The panel also highlighted the value of holistic care for the management of Long COVID.

**Conclusion:**

Long COVID is a major public health challenge. These expert recommendations are expected to guide healthcare professionals in their clinical decision-making for the assessment, management, and appropriate rehabilitation of patients with Long COVID.

## 1 Introduction

Globally, the coronavirus disease 2019 (COVID-19) pandemic has resulted in a considerable amount of illness and mortality. Although COVID-19 largely affects the respiratory system, data show that it is a severe multisystem illness and often results in fatal consequences (1). Many patients with COVID-19 recover within a few days to weeks; however, symptoms may persist weeks to months in some patients, severely affecting the quality of life (QoL), long after the acute COVID-19, commonly referred to as post-COVID, post-acute sequelae of COVID-19 or Long COVID. Long COVID patients experience a broad range of symptoms (above 100 symptoms reported), which can continue or reappear after acute respiratory syndrome coronavirus 2 (SARS-CoV-2) infection, such as fatigue, dyspnea, olfactory and gustatory impairments, insomnia, cognitive disorders, or mental implications, such as anxiety and depression. In some patients, the condition may be associated with damage of the heart, lungs, or liver (2–9). Long COVID can affect all age groups, at varying degrees of severity (10–13). Studies across the world demonstrate that Long COVID condition is connected with a poor QoL, and the relapsing–remitting persistent symptoms can negatively impact physical and cognitive function, and participation in society (14–16).

Several studies have demonstrated persistence of COVID-19 symptoms over a long period. However, data on Long COVID are highly heterogeneous, demonstrating the differences in definitions, the cohorts examined, and follow-up durations (17). In the year 2022, a meta-analysis estimated that pooled worldwide prevalence of post COVID-19 condition (persistence of symptoms ≥28 days following diagnosis) as 0.43 (95% CI: 0.39, 0.46), while the hospitalized and non-hospitalized patients have estimates of 0.54 (95% CI: 0.44, 0.63) and 0.34 (95% CI: 0.25, 0.46), respectively (18). As of March, 2022, based on the World Health Organization (WHO)'s estimation of 470 million COVID-19 infections, approximately 200 million individuals currently experience or have previously experienced long-term consequences of COVID-19 (18). Another analysis that defined Long COVID as new onset or persisting symptoms ≥3 months after diagnosis, estimated that 144.7 million (95% CI: 54.7, 312.6) cases across the globe suffered from one to three common symptoms such as fatigue, cognitive problems and respiratory problems of Long COVID, during 2020 and 2021. Among these patients, 15.1% (95% CI: 10.3, 21.1) patients continued to have persistent symptoms beyond 12 months of COVID-19 infection (19). A recent review estimated that globally around 65 million people may have long COVID, based on an analysis of 10% of infected people and >651 million COVID-19 cases worldwide (20).

Children’s Long COVID prevalence ranged between 4 and 66% (21). Additionally, a global systematic review projected that 25.24% of children and adolescents suffer from ≥1 symptoms for more than 4 weeks following a SARS-CoV-2 infection and the prevalence among hospitalized children and adolescents was slightly higher (29.19%) (22). Reinfection, which is defined as “second positive SARS-CoV-2 test ≥90 days after the first positive test,” is yet another factor that could augment the increased prevalence of Long COVID’ (23). However, there are limited studies that have assessed disease burden of reinfection. As per few publications in Saudi Arabia, severe illness leading to intensive care unit admission and death in reinfected people is rare and principally occurs in the unvaccinated group (24, 25).

Although limited, currently available data suggest that the burden of Long COVID is substantial in the region (6, 26–29). An online cross-sectional survey (*n =* 5,946), showed that 79.4% have unresolved symptoms for at least 4 weeks following the disease onset (26). Furthermore, 9.3% were hospitalized with 42.7% visiting healthcare facility after discharge and 14.3% requiring readmission (26). A national representative study (*n =* 1,000) from COVID-19 patients registered at the Saudi Ministry of Health (MOH) Database (*N =* 314,821) demonstrated 22.5% of patients showing late symptoms (beyond 4 weeks following diagnosis) (27). A web-based cross-sectional study from Saudi Arabia, reported that 35.5% (*n =* 972) and 38.8% (*n =* 1,063) of patients showed persistent COVID symptoms at 4–6 weeks and 6 weeks to 6 months after disease onset, respectively (28). Local data are required to draw conclusions and survey data need to be assessed cautiously due to subjective nature of surveys that might be influenced by recall bias and the differences in defining Long COVID make the epidemiological comparison challenging.

Several studies are being conducted around the world (30) (e.g., RECOVER and LONG COVID RESEARCH INITIATIVE etc.) to better understand Long COVID (31–34). Despite the mounting evidence, there is a substantial knowledge gap in terms of pathophysiology, disease epidemiology, risk factors, diagnosis and management of Long COVID. At present, there are no clear diagnostic criteria for Long COVID and there is no consensus on the algorithm of investigations and clinical management of symptoms in the Middle East region.

Locally developed guidelines for the management of Long COVID are available (e.g., Ministry of National Guard Health Affairs) (35, 36); however, there is a need to address gaps in the comprehensive and holistic management based on the ever-evolving international best practices. An expert panel was formed to address the current gaps in defining Long COVID, understanding the disease and formulate expert management recommendations from the region’s perspective. This article presents the expert recommendations from a secondary desk research and the expert insights from the virtual advisory board meetings conducted in Saudi Arabia.

## 2 Methods

A group of 10 expert consultant physicians was formed to develop the current recommendations for the management of Long COVID with a focus on the region’s perspective. The panel represented multidisciplinary specialties involved in the management of both acute and Long COVID.

A secondary desk research was conducted to address unmet needs in Long COVID care. However, capturing the relevant information during the secondary desk research was challenging as there are limited data on Long COVID pertaining to the region. Nevertheless, key findings from the available literature were presented in the advisory board meetings. The expert panel reviewed the current international guidelines and literature to identify the gaps in literature. Three virtual advisory board meetings were held to generate the recommendation statements regarding the management of Long COVID, based on the current evidence and their expertise.

The discussion mainly involved epidemiology, magnitude of the problem, disease pathogenesis, diagnosis, prevention and management. The key objectives of the expert panel meetings were as follows:

1. To critically review the available literature and international recommendations on the management of patients with Long COVID, identify gaps in defining Long COVID and highlight unmet needs in current clinical practice.
2. To develop regional recommendations for the effective management of Long COVID.

## 3 Results and discussion

### 3.1 Definition of Long COVID

“Long COVID condition” is an umbrella term and several terms have been used to describe prolonged symptoms following COVID-19, such as “Long COVID,” “postacute sequelae of SARS-CoV-2 infection (PASC),” “postacute COVID-19,” “chronic COVID-19,” and post-COVID syndrome’ (37). A globally acceptable scientific definition is yet to be established. The limited scientific evidence and the methodological differences among the available studies make it challenging to study Long COVID.

The WHO defines “Post COVID condition or Long COVID as the continuation or development of new symptoms 3 months after the initial SARS-CoV-2 infection, with these symptoms lasting for at least 2 months with no other explanation” (38). The Centers for Disease Control and Prevention (CDC) (37) defines that post-COVID conditions (PCC, or Long COVID) are a wide range of new, returning, or ongoing health problems people can experience four or more weeks after first being infected with the virus that causes COVID-19. The National Institute for Health and Care Excellence (NICE) guidelines defined the condition as “ongoing symptomatic COVID-19, if signs and symptoms persisted from 4 to 12 weeks and as post-COVID-19 syndrome, if signs and symptoms continued beyond 12 weeks and are not explained by an alternative diagnosis (39).” A collection of proposed definitions of Long COVID is presented in the WHO’s review on clinical case definition of post-COVID condition (40).

Lack of a standard definition and consistent terminology for Long COVID, limits researchers from studying the condition, its clinical diagnosis and treatment. Therefore, the current expert panel agreed on the following definitions, considering all the deficiencies in the current case definitions.

*Post-acute COVID:* persistent or new COVID-19 related symptom/s between 3 and 12 weeks after confirmed diagnosis [confirmed either by antigen test or reverse transcription polymerase chain reaction (RT-PCR)].

*Long COVID syndrome:* persistent or new COVID-19 related symptom/s beyond 12 weeks from confirmed diagnosis (confirmed either by antigen test or RT-PCR), irrespective of age, which cannot be explained by an alternative diagnosis, impacting QoL, which may or may not be associated with end-organ damage.

### 3.2 Pathophysiologic mechanisms

Current literature shows that Long COVID is multisystemic in nature and further research on its pathophysiologic mechanisms of Long COVID are warranted. However, potential risk factors may include acute SARS-CoV-2 injury, persistence of SARS-CoV-2 in certain tissues, reactivation of neurotrophic pathogens such as herpesviruses, immune dysregulation, SARS-CoV-2 interplay with host microbiome/virome communities, clotting/coagulation issues, anomalous brainstem/vagus nerve functioning, activity of primed immune cells, and autoimmunity due to molecular mimicry between pathogen and host proteins (4, 41, 42). The promiscuous nature of Long COVID makes it affect multiple organs, including pulmonary, neuropsychiatric, cardiovascular and gastrointestinal systems. SARS-CoV-2 binds to angiotensin-converting enzyme 2 (ACE2) receptors, abundantly expressed in various tissues. This binding facilitates viral entry into cells, leading to dysfunction and disruption of normal physiological processes (43). The virus’s ability to evade the immune system and dysregulate the host immune response contributes to hyperinflammation and elevated production of proinflammatory cytokines (42, 44). Additionally, mechanisms such as endothelial damage, activation of clotting cascades, and the development of microvascular dysfunction and microthrombi further contribute to the disease’s systemic effects (42, 45).

The respiratory system has been impacted, with evidence of airway epithelial damage, local inflammation, and inflammatory cell infiltration in the lungs (42, 46). The nervous system has been affected through mechanisms such as neuroinflammation, neuronal injury, blood vessel coagulopathy, and endothelial damage (45). Additionally, mitochondrial dysfunction and abnormal levels of mitochondrial and SARS-CoV-2 proteins in the central nervous system have been reported (47).

The reproductive system has also shown significant impacts in both men and women. In women, long COVID has been associated with menstrual irregularities, gynecological conditions, and fertility-related issues (48). The virus infiltrates ovarian cells via ACE2 receptors, disrupting ovarian function, hormone levels, follicular development, oocyte maturation, and embryo implantation. Psychological stress from infection affects the hypothalamic–pituitary–adrenal (HPA) and hypothalamic–pituitary-gonadal (HPG) axes, leading to hormonal dysregulation and menstrual disruptions (48). Estrogen, cortisol, and immune response interactions play key roles. In men, SARS-CoV-2 affects testicular cells with high ACE2 expression, induces secondary immune responses and inflammation (49), and impacts the hypothalamus-pituitary-gonad axis (50). The virus has also been linked to erectile dysfunction caused by endothelial damage (51) and impaired sperm count, motility, and concentration associated with elevated cytokine levels (52).

The gastrointestinal and biliary systems have been impacted by significant alterations in gut microbiota, with an increase in opportunistic pathogens (53). SARS-CoV-2 has been detected in stool samples for up to 4 months post-diagnosis (54), and antigen persistence in long COVID cases, especially with inflammatory bowel disease, has been observed for up to 7 months (55). The cardiovascular system has been affected through direct myocardial inflammation caused by the virus, regardless of disease severity (42). Coagulopathy, immunothrombosis, and hyperinflammation associated with COVID-19 further contribute to cardiovascular complications (56).

The endocrine system is also impacted, primarily through the hypothalamic–pituitary axis, with both direct and indirect cellular injury leading to hormonal dysregulation and functional impairments (50).

The reactivation of latent viral infection, particularly herpesviruses has been discussed to be linked to virus illness related stress and inflammation rather than a direct cause-effect relationship. Its emergence in association with COVID-19 infection warrants further analysis to better understand this coincidental occurrence (57).

**Figure 1:**
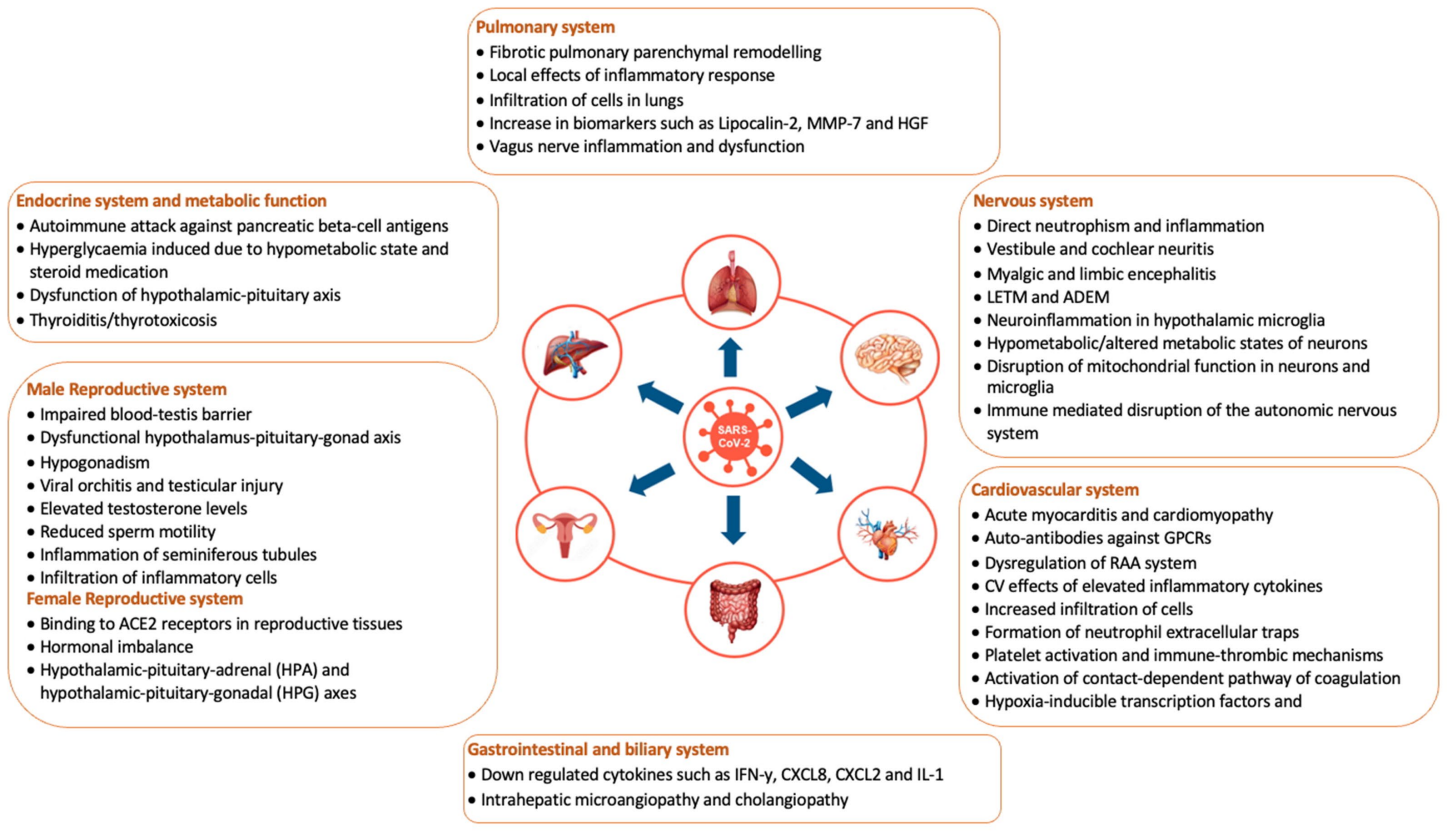
Organ specific pathophysiological mechanisms in Long COVID. ADEM, acute disseminated encephalomyelitis; CV, cardiovascular; CXCL8, C-X-C motif ligand 8; GPCRs, G-protein-coupled receptors; HGF, hepatocyte growth factor; IFN-*γ*, interferon-gamma; IL-1β, interleukin-1-beta; LETM, longitudinally extensive transverse myelitis; MMP-7, matrix Metalloproteinase-7; RAA, renin-angiotensin-aldosterone system. Sources: Davis et al. (20), Proal et al. (42), Saunders et al. (58), and Umesh et al. (50).

Figure 1 depicts organ-specific pathophysiological mechanisms in Long COVID. The expert panel concurred with the currently available literature that Long COVID is multisystemic and deeply in association with anomalous immune system. Further, the expert panel also opined that the principal mechanisms behind the Long COVID symptoms could be chronic inflammation, specific organ damage, damage to blood vessels, resulting in blood clots and presence of lingering viral particles in the blood or tissues.

### 3.3 Symptoms

Long COVID may occur with diverse debilitating symptoms and conditions, which varies with individuals, and the degree of severity may range from disturbances of wellbeing to massive limitation of daily life. Some patients recover within weeks or months after the illness, while some experience relapses. Symptoms may follow a relapsing–remitting pattern, with recurrent spikes of fever reported in previous studies (59–61). The persistence of symptoms may also vary with the COVID-19 variant (62, 63). Across various systematic reviews the most common symptoms of Long COVID are fatigue (31–58%), dyspnea (24–40%), insomnia (11–44%), mental anomalies (or brain fog; 12–35%), anosmia (10–22%), musculoskeletal pain (9–19%), cough (7–29%), and chest pain (6–17%) (2, 64–69).

Incidence of specific symptoms may vary with observation period and severity of the infection; as per a global systematic review on prevalence of Long COVID symptoms the most frequent symptoms were fatigue, dyspnea, sleep disorder, and difficulty concentrating (32, 25, 24, and 22%, respectively, at 3- to <6-month follow-up); effort intolerance, fatigue, sleep disorder, and dyspnea (45, 36, 29, and 25%, respectively, at 6- to <9-month follow-up); fatigue (37%) and dyspnea (21%) at 9 to <12 months; and fatigue, dyspnea, sleep disorder, and myalgia (41, 31, 30, and 22%, respectively, at >12-month follow-up) (70). Most frequently reported symptoms of pediatric Long COVID include fatigue, headaches, stomach/abdominal pain, muscular aches, post-exercise malaise, and rashes (22, 71, 72).

A latest study based on the data (*n =* 5,946 patients) from the Saudi MOH COVID-19 testing registry, reported that fatigue (53.5%), muscle and body pain (38.2%), anosmia (35.0%), joint pain (30.5%), and ageusia (29.1%) were the persistent symptoms beyond 1 month following diagnosis (26). Another study from Saudi Arabia that monitored the patients (*n =* 222) from 6 weeks to 6 months post hospital discharge, demonstrated that dyspnea (40.1%), cough (27.5%) and fatigue (29.7%) were the frequent symptoms at follow-up (29). Persistent symptoms and functional ability, including organ damage, seem to substantially affect patients’ QoL and to daily activities. A global systematic review (39 studies) found impaired QoL among 57% of patients with symptoms persisting beyond 12 weeks following diagnosis (73).

Acute respiratory symptoms generally resolve over time, whereas neuropsychiatric conditions often emerge later and persist for longer durations. The predominant focus on respiratory symptoms as the primary sequelae of SARS-CoV-2 infection has distracted efforts to identify and manage other long-term impacts, including neurobehavioral, cardiovascular, and effects on other body systems (20, 51). Further research is needed to quantify these observations, considering underlying molecular mechanisms, the severity of acute illness, reinfection, vaccination status, comorbidities and other host-related risk factors.

### 3.4 Risk factors

Data on potential risk factors for Long COVID symptoms are accumulating, though they are heterogeneous. Common risk factors for Long COVID are severity of acute COVID-19, female gender, ethnic minority, socioeconomic deprivation, smoking, obesity, and a wide range of comorbidities (11, 74–76). It is projected that women have two-fold risk of having Long COVID symptoms (OR: 1.3–5). Similarly, severe acute COVID-19 has been associated with an elevated risk for Long COVID symptoms, with the strongest association with fatigue (76).

A US based study (*n =* 309) found that the presence of certain autoantibodies, reactivation of the Epstein–Barr virus (EBV), and type 2 diabetes are the major risk factors for Long COVID (77). A retrospective cohort study, from the US Department of Veterans Affairs health care system found that older age, Black or American Indian/Alaska Native race, Hispanic ethnicity, geographical region, high Charlson Comorbidity Index score, having documented symptoms at the time of acute infection (adjusted OR: 1.71; 95% CI: 1.65, 1.78) and requiring hospitalization (AOR: 2.60; 95% CI, 2.51–2.69) or mechanical ventilation (adjusted OR, 2.46; 95% CI: 2.26, 2.69) were significantly associated with Long COVID (78). In another US based study among children and adolescents (aged <21 years; *n =* 358), who were hospitalized for acute COVID-19 or multisystemic inflammatory syndrome (MIS-C), >1 in 4 patients had persistent symptoms or anomalous activity after having 2–4 months to recover from the illness. The study found that patients with MIS-C and respiratory comorbidities or obesity are at a risk of slow recovery (79).

In an Iran based study of Long COVID patients (*n =* 4,681), a shorter hospital stay was inversely linked with Long COVID syndrome (OR: 0.953; 95% CI: 0.941–0.965; *p* = 0.0001) (80). In Saudi Arabia, an observational study (*n =* 7,520) found that female gender, old age, multiple comorbidities, long-term medications, length of hospital stay, and tenure of acute COVID-19 symptoms could be important predictors of post-COVID-19 (28). A national representative study from Saudi Arabia (*n =* 1,000), found that the presence of acute symptoms of COVID-19 and hospital admission were significant independent predictors of post-COVID-19 condition (OR: 15.0, 95% CI: 2.1, 109·4; *p* = 0.008 and OR: 2.3, 95% CI: 1.3, 3.9; *p* = 0.002, respectively) (27).

The risk factors for long COVID are multifaceted, involving demographic, clinical and biological variables. Factors such as disease severity, female gender, comorbidities and socioeconomic disparities are consistently reported across studies, while additional contributors such as autoantibodies, viral reactivation, and prolonged inflammation/immune dysregulation have emerged as significant.

### 3.5 Impact of viral strain variance on Long COVID

Strain variance and cofactors can impact severity of Long COVID and the effectiveness of COVID-19 vaccination. There is paucity of data on the association of viral strain/variant with Long COVID. Several studies are currently exploring the association of specific viral strains with the incidence and magnitude of Long COVID symptoms. In a case–control observational study from the UK, Long COVID was more prevalent in the people infected with Delta variant (10.8% [4,469/41361]) compared with the people infected with Omicron variant 4.5% (2,501/56003) (81). Patients with Omicron variant were less likely to have Long COVID, with an odds ratio ranging from 0.24 (0.20–0.32) to 0.50 (0.43–0.59) (81). Similar findings were noted in a latest India based study (*n =* 524), which found that self-reported Long COVID (persistence of COVID related symptoms for ≥28 days after diagnosis) is considerably low among COVID-19 patients diagnosed during the Omicron wave versus Delta wave in the same setting (82). As per an exploratory survey in the US, frequency of Long COVID symptoms was similar by predominant variant at the time of illness, with the exception that anosmia was less frequently presented for infections when the Omicron variant was the predominant variant (Omicron variant, 83 of 246 [33.7%]; Alpha variant, 59 of 147 [40.1%]; Delta variant, 210 of 416 [50.5%]; *p* < 0.001) (83).

A China study found that people infected with the Wuhan variant had a more number of symptoms (higher prevalence of fever, dyspnea or gastrointestinal problems) than those infected with Alpha or Delta variant (*p* < 0.01) (84). The mean number of post-COVID-19 symptoms was higher (*p* < 0.001) in individuals infected with the Wuhan variant (mean: 2.7 ± 1.3) than those with the Alpha (mean: 1.8 ± 1.1) or Delta (mean: 2.1 ± 1.5) variants (84). Post-COVID-19 dyspnea was more prevalent (*p* < 0.001) in people infected with the Wuhan variant, whereas hair loss was greater in those infected with the Delta variant (*p* = 0.002). A higher proportion of patients infected with the Delta variant reported headache, anosmia or ageusia as onset symptoms (*p* < 0.01) (84).

The expert panel mentioned that children and adult patients showed different symptoms with the infected strain (specifically in patients with comorbidities). Further, the panel added that Omicron variant was more infectious in children; other strains were mildly infectious, treated symptomatically and only a few patients required hospital admissions; the panelists found that majority of the children who required admission were infected with the Omicron strain. The experts also mentioned Omicron BA.4 and BA.5 were more contagious and caused several breakthrough infections.

### 3.6 Assessment of Long COVID

*Expert recommendations*

- Upon the first encounter of a patient with ongoing symptomatic COVID-19, a thorough clinical history and appropriate assessment should be performed to evaluate functional abilities as well as physical, cognitive, psychological, and psychiatric symptoms. COVID-19 Yorkshire Rehabilitation Scale (C19-YRS) and Newcastle Post-COVID Screening Tool are the common questionnaires for initial screening of patients with Long COVID
- The typical clinical evaluation can include history of acute COVID-19 (suspected/confirmed), the magnitude of symptoms, and persistence of symptoms since the attack of acute COVID-19, history of comorbidities, exacerbation of comorbidities, current and previous drug treatment/s (including unprescribed medications, herbal remedies, supplements, or any other treatments)
- In the first encounter, if the person/patient reports/shows cognitive/psychological symptoms, a validated screening tool should be used to measure any impairment and impact, with particular attention to the onset of symptoms of anxiety, depression, post-traumatic stress disorder (PTSD) and social isolation. The Hospital Anxiety and Depression Scale, the Patients Health Questionnaire 9, the General Anxiety Disorder 7, or the Depression Anxiety Stress Scale 21 and the Montreal Cognitive Assessment for a cognitive screening are the commonly used tools to assess neuropsychiatric symptoms
- The basic diagnostic tests should be personalized according to the patient’s signs and symptoms to rule out acute or life-threatening complications and find out if symptoms are likely to be the result of Long COVID or unrelated diagnosis. Depending on the clinical presentation of the patient, basic diagnostic investigation can include the following tests (but is not limited to):
- General blood tests: complete blood count (CBC), haemoglobin A1c (HbA1c), random blood glucose, C-reactive protein (CRP), erythrocyte sedimentation rate (ESR), ferritin, nutritional profile (vitamin D, vitamin B12 and folate), renal profile, liver profile, thyroid function, creatine phosphokinase, troponin, B-type natriuretic peptide (BNP), and D-dimer
- Systemic evaluation and other tests include administration of screening questionnaire for Long COVID and psychological/psychiatric symptoms (PTSD/anxiety/depression etc); physical examination; vital parameters; Chest X-ray; electrocardiogram (ECG)
- Pediatrics assessment can include a stepwise approach, with initial conservative evaluation to focus on determining level of symptom interference with daily functioning, enforcing, and aiding a return to healthy lifestyle habits (sleep, diet, light activity as tolerated without symptom exacerbation) and ruling out other causes of ongoing symptoms
- Initial tests can include CBC with differential and comprehensive metabolic panel (CMP), CRP, ESR, ferritin, thyroid-stimulating hormone (TSH), and vitamin D level
- If symptoms are affecting the patient’s daily routine activities, then additional diagnostic tests or referral to consultation with a multidisciplinary pediatric post-COVID-19 clinic should be considered.
- If a multidisciplinary pediatric post-COVID-19 clinician is unavailable, consider referral to a pediatric medical subspecialist

Long COVID condition presents a wide range of symptoms and conditions, and its appropriate management begins with a comprehensive assessment of an individual’s clinical and functional status. Upon the first encounter of the patient, the healthcare professionals should check for emergency or life-threatening complications (e.g., acute respiratory insufficiency, or cardiogenic chest pain etc.) and refer the patient to acute services for further care.

If the patient presents with stable and non-severe symptoms, respective healthcare professionals should perform primary evaluation including a detailed patient history, the examination of persistent physical, cognitive, psychological, and psychiatric symptoms, and comorbidities. Furthermore, the primary assessment should consider the premorbid conditions (e.g., malignancy), other differential diagnoses (e.g., thromboembolic events, myopericarditis, encephalitis) as well as the socioeconomic circumstances of the patients.

Clinical presentation in children is varied and hence, the physician needs to assess whether the child is totally healthy, has comorbidities, has any genetic predisposition or severe COVID-19 etc. It is advisable to prescribe minimal investigations and include only basic tests for general evaluations.

**Figure 2:**
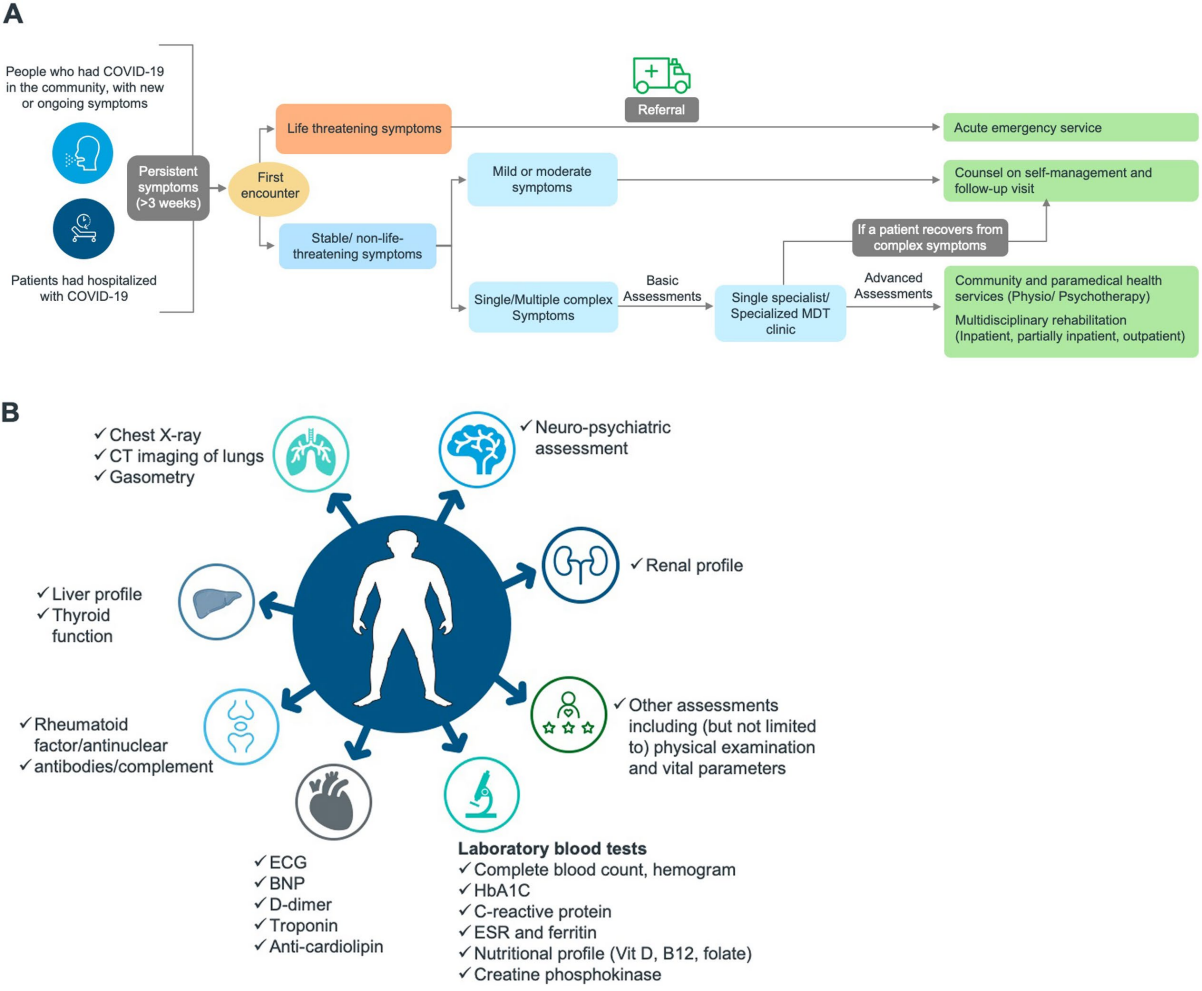
Proposed care pathway for Long COVID in all age groups. **(A)** Management pathway. **(B)** Diagnostic landscape (basic and advanced assessments). BNP, B-type natriuretic peptide; COVID-19, Coronavirus disease 2019; ECG, electrocardiogram; ESR, erythrocyte sedimentation rate; HbA1C, hemoglobin A1C/glycated haemoglobin; MDT, multidisciplinary team.

Generally, there can be similarities as well as differences/variations with respect to the spectrum of presentation of a patient. The expert panel opined that the following general blood tests and other evaluations can be prescribed for symptomatic patients according to the physician’s discretion (Figure 2B):

General blood tests including complete blood count, haemogram, HbA1c, random blood glucose, C-reactive protein, ESR, ferritin, nutritional profile (vitamin D, vitamin B12, folate), renal profile, liver profile, thyroid function, creatine phosphokinase, troponin, BNP, D-dimer and procalcitonin (PCT). Systemic evaluation and other tests include administration of screening questionnaire for Long COVID and psychological/ psychiatric symptoms (post-traumatic stress disorder [PTSD]/anxiety/depression etc.,); physical examination; vital parameters; chest X-ray and ECG. Primary care centers can use various questionnaires and scales, for their diagnosis, including (but is not limited) (39, 85, 86):

- The Newcastle Post-COVID Syndrome Follow-up Screening Questionnaire for a detailed face to face multi-disciplinary evaluation if symptoms continue for 10–12 weeks following the diagnosis (87).
- The COVID-19 Yorkshire Rehabilitation Screening Tool to screen problems related to the recent illness with COVID-19 (88).
- The EQ-5D and the Short-Form 36 to assess the generic health status of the patients (89, 90).
- The Post-COVID-19 Functional Status Scale (PCFS) Scale to assess Long COVID-related individual distress and level of anomalies (91).
- The Hospital Anxiety and Depression Scale (92), the Patients Health Questionnaire 9 (93), the General Anxiety Disorder 7 (94) or the Depression Anxiety Stress Scale 21 (95) and the Montreal Cognitive Assessment for a cognitive screening (96).

In primary healthcare centers patients can also be inquired whether the patients are administering any unprescribed medications, herbal medications, supplements, or other treatments. In the subsequent encounter of a symptomatic patient at a healthcare facility, relevant advanced diagnostic tests can be prescribed such as gasometry (baseline oxygen saturation is persistently decreased), computed tomography (CT) imaging of lungs as per the x-ray findings.

To overcome the barriers in evaluating patients for Long COVID, the expert panel suggested improving access to care, training primary care physicians, accommodating/allocating the manpower for finding the Long COVID cases, performing initial assessment, referral, and support with the budgetary needs, developing a simple tool to create awareness about Long COVID, ensuring early diagnosis, using applications for finding the Long COVID cases and linking them for care.

### 3.7 Role of primary care in the management of Long COVID

Primary healthcare is pivotal in managing Long COVID as it includes conducting initial assessment, triaging and identifying the patients who are unable to link their symptoms to COVID-19 (previously confirmed or suspected), investigating and excluding acute, post-acute or life-threatening conditions and other unrelated diagnoses prior to refer them to post-COVID services, prescribing investigations in relation to symptom presentation in line with the standard guidelines supporting follow-up care and rehabilitation. Effective triage and management of symptoms at a primary care center may eventually help reducing disease burden at secondary and tertiary care centers.

The expert panel highlighted the need for improved knowledge and skills of primary healthcare personnel to differentiate the patients by their symptoms (new or evolving) clinical presentation, managing the underlying diseases such as uncontrolled diabetes, uncontrolled cardiac or psychiatric conditions etc. Setting up targeted workshops to increase the awareness and skills of primary care personnel about Long COVID and other underlying comorbidities and introducing virtual communication platforms can revolutionize the training of primary healthcare professionals. The panel also suggested a programme coordinator to oversee the activities on a whole and communicate as well as guide the patient journey through primary centers to referred specialized centers.

### 3.8 Long COVID healthcare pathway

Upon reviewing the findings of secondary desk research on Long COVID care pathways from the various published guidelines, the panel opined that the recommendations and the implemented services might differ among countries (97–101). Self-management and primary care are the main support in Long COVID healthcare, accompanied by specialist care, non-clinical therapies, and rehabilitation if necessary. Additionally, a multidisciplinary and patient-centred approach and empathy toward the patients and caregivers were the key factors suggested for successful Long COVID care. Finding the right balance between required treatments is important to prevent uncertainty and anxiety among patients. Overall, Long COVID management pathway is a complex interaction of various systems. Therefore, it encompasses coordinating support from across the fragmented systems and guaranteeing care that caters the individual needs of patients and their families. The expert panel came up with a holistic Long COVID care pathway, considering the local requirements and infrastructure in Saudi Arabia and the region (Figure 2A).

### 3.9 Management of symptoms

*Expert recommendations*

- Refer patients urgently to the relevant acute services if they have signs or symptoms due to an acute or life-threatening complication, including (but is not limited to):
- Severe hypoxaemia or oxygen desaturation on exercise
- Signs of severe lung disease
- Cardiac chest pain
- Multisystem inflammatory syndrome (in children)
- Based on the basic investigations and after ruling out serious or fatal complications and other diagnoses, if the patient is stable, advise self-management, and one of the following, depending on clinical need:
- To self-manage their symptoms
- Whom to consult to manage their symptoms or to support with self-management
- Sources of advice and support, including support groups, social prescribing, online forums, and applications
- Referral to an integrated multidisciplinary assessment or referral to specialist care
- Increase the awareness on COVID-19 vaccination and encourage the people to vaccinate as per the current official recommendations to lower their probability of contracting another acute infection. Explain the impact of vaccination on Long COVID, which warrants further research
- Some of the additional required vaccines may be recommended (e.g., influenza and pneumococcal vaccines) based on age or risk factors including chronic illness or lung affection following acute COVID. Herpes zoster recombinant vaccine can be suggested for people who are ≥50 years of age
- Treatment should be consistent with the current standard guidelines in the respective field; current COVID-19 guidelines from the Saudi MOH, should be adopted, as applicable in managing COVID-19 specific symptoms
- Rehabilitation plans need to be individualized according to the patient’s needs and comorbidities
- Patients should be sensitized on their condition and its management until recovery
- If warnings, clinical deterioration, or ambiguities are seen in the basic diagnosis, those affected should be offered in-depth advanced diagnosis and/or referred to suitable specialist disciplines
- Psychosomatic basic care is suggestible at an early stage for the symptoms such as history of similar somatic or psychosomatic complaints, high psychosocial burden or earlier frequent consultations with fruitless somatic diagnostics
- Access to care can be improved by utilizing all the available data from the government portals and repurposing the digital applications such as TAWAKKALNA and SEHHATY for the management of Long COVID
- Patients with severe/critical illness should be assessed for medical fitness before return to work. QoL assessment is advised prior to returning to work. Life-style modifications (nutrition and exercise) needs to be general and tailored to the patient, based on the residual symptoms
- The recommendations on life-style modifications (nutrition and exercise) needs to be general and tailored to the patient based on the residual symptoms.
- QoL assessment before going to work would be very helpful.

Despite the advent of several guidelines on the management Long COVID, there is still a significant practical gap in customizing symptom-based treatments for patients. A thorough analysis of the present clinical strategies used by clinicians will be helpful in directing the proper patient rehabilitation (97, 102, 103). The panel opined that finding the patients with Long COVID and approaching them for care and building a registry are very critical in holistic management; further, improvement in the community awareness regarding Long COVID and following up of the patients on the symptoms would make management better and reduces the disease burden. The guidelines for management of Long COVID will be dynamic nature; however, present expert recommendations may be useful in managing Long COVID, particularly within the region.

### 3.10 Fatigue

Fatigue is the commonest of all the symptoms reported in patients with Long COVID (26, 29, 70, 104), similar to community acquired pneumonia, severe acute respiratory syndrome (SARS), Middle East respiratory syndrome coronavirus (MERS), and Ebola epidemics (97). A recent global meta-analysis (*n =* 63 studies with *n =* 257,348 COVID-19 patients), found fatigue to be the most common symptom during follow-up periods, with a prevalence of 32% at 3- to <6-month follow-ups, 36% at 6- to <9-months, 37% at 9 to <12 months and 41% at >12-month follow-up (70). A latest study from Saudi Arabia (*n =* 5,946 patients) showed 54% prevalence of fatigue at 1 month of COVID-19 diagnosis (26). In another study (*n =* 1,000), prevalence of fatigue was found to be 11.5%, at 12 weeks after the onset of acute COVID-19 (27).

Fatigue can be developed either during acute phase or developed after recovery from the acute phase of infection. Cytokines produced during SARS-CoV-2 infection may affect psychological defense mechanisms, pre-existing autoimmune conditions, and increased ANA (antinuclear antibodies) are the factors that can cause fatigue. There is also a probable link between premorbid depression/anxiety and post-COVID-19. A recent systematic review determined that the most predisposing factors for post COVID fatigue can be old age, female gender, severe clinical condition during acute phase, more comorbidities, premorbid depression/anxiety, hospital admission and oxygen supplementation at the acute phase (105). Further, psychological and social factors such as helplessness in illness, avoidance behaviors, financial worries due to unemployment and loneliness due to limitations in social contacts could be associated with the development of fatigue following COVID-19 (106).

Presently, there is a lack of evidence to support pharmacological or non-pharmacological interventions for the management of fatigue in the patients with Long COVID (97, 105). Complementary and alternative medicine, cognitive behavioral therapy, and exercise were commonly used to manage fatigue (107). Also discussion on the role of graded exercise and cognitive behavioral treatment in chronic fatigue is underway (108, 109). Patient resources on fatigue management (110) and guidance for clinicians on return to exercise (111) and graded return to performance for athletes (112) in COVID-19, are based on indirect evidence. The expert panel suggested promoting gradual physical activity for patients with Long COVID; however, if the patient develops breathlessness, severe fatigue or muscle aches, medical consultation is required.

### 3.11 Cough

Persistent cough is a common symptom of COVID-19 in the first 6–12 weeks after the acute illness. In global studies, long-term cough was observed in 33–43% of cases at 4 weeks (60, 113), in 5–46% at 8 weeks (114–116), and in 2–17% at 12 weeks (117, 118). Whereas in Saudi Arabia, it was reported in 27.5% of patients at 6 weeks (29), in 8.9–47% at 12 weeks (8, 27) after the onset of acute COVID-19.

Presently, there is a lack of studies assessing the treatment of persistent cough after acute COVID-19. Simple breathing exercises and medication, when necessary, are the best ways to treat cough, unless there is super-infection or other complications, such as unpleasant pleural inflammation. In general, inhaled corticosteroid (ICS) and/or beta-2 sympathomimetics are used for post-infectious cough (119), particularly if there is bronchial hyperreactivity; proton pump inhibitors are prescribed if reflux is suspected (97). A recent review on the pathophysiology and management of cough in patients with COVID-19, suggested further assessment of gabapentin and pregabalin, antimuscarinic drugs, and other novel drugs for their application in persistent cough (66).

### 3.12 Dyspnea

Dyspnea refers to shortness of breath and strain in breathing. Breathlessness is common among patients with Long COVID. In global studies, long-term dyspnea was observed in 11–33% of cases at 4 weeks (60, 113, 120), in 8–63% of cases at 8 weeks (114–116, 120), and in 14% beyond 12 weeks (117). In Saudi Arabia, long-term dyspnea was reported in 10.2% of patients at 4 weeks (27), in 40.1% of patients at 6 weeks (29), in 6.9% of patients at 8 weeks (7) and in 40% beyond 12 weeks (8) after the onset of acute COVID-19.

Acute COVID-19 may significantly impair the lungs and respiratory tract via SARS-CoV-2 replication in the endothelial cells, resulting endothelial damage and vigorous immune and inflammatory reaction (121, 122). People who are recovering from acute COVID-19 may develop long term anomalies in lung function, leading to dyspnea (123); however, majority of the people who develop long-term breathing problems post COVID-19 present no signs of permanent or long-lasting lung damage (113, 124). It seems that only the older people, those who endure acute respiratory distress syndrome, have prolonged hospital stays, and have pre-existing lung anomalies, are susceptible to fibrotic-like changes to lung tissue (125). The fibrotic state found in some patients with ongoing dyspnea may be stimulated by cytokines such as interleukin-6, which increases COVID-19 infection (126) and is involved in the formation of pulmonary fibrosis (127). Pulmonary vascular thromboembolisms have been observed in patients with COVID-19 and may have detrimental consequences in patients with Long COVID (128). Breathlessness tends to improve with breathing exercises. Severe breathlessness is rare, but it may require urgent referral (129). In general, physicians prescribe inhaled corticosteroids or betamimetics for the patients, if there is evidence of respiratory obstruction (130).

### 3.13 Chest pain

Chest pain is frequent in patients with Long COVID. Chest pain can be expressed as any pain located between the diaphragm and the base of the neck. Chest pain may exist in 20% of patients at 4 weeks (60), 22% at 8 weeks (115) and 11% at 12 weeks (118). For the management of chest pain, the clinical priority is to segregate musculoskeletal and other kind of non-specific chest pain from serious cardiovascular conditions. Clinical assessment of the post-acute COVID-19 patient with chest pain should be adhered to the principles to that for any chest pain (111). When the diagnosis is uncertain, or the patient is severely unwell, urgent cardiology referral should be considered for specialist assessment and investigations (97).

### 3.14 Cardiovascular abnormalities

Cardiovascular complications are common in patients with Long COVID. Persisting cardiovascular anomalies can be difficult for people with Long COVID (131). An analysis from the US reported a huge burden of cerebrovascular disorders, arrhythmia, ischaemic heart disease, heart failure, and thrombotic disorders between 30 days and 12 months after COVID-19 infection versus to the control cohorts (132). Cardiac injury and high cardiac troponin levels were associated with a significantly greater risk of mortality in patients hospitalized with acute COVID-19 infection (133). Additionally, autonomic dysfunction, especially manifesting as postural (orthostatic) tachycardia syndrome (PoTS), occurs commonly in post COVID-19 condition (134). Patients with Long COVID recovering from cardiac injury, with functional limitations, can undergo cardiac rehabilitation rather than a traditional physical therapies, if there are no contraindications. PoTS treatment can be started with fluids, compression, and lifestyle adaptations (135), but it can be changed to medication if symptoms are persisting (136, 137).

Some patients with Long COVID may demonstrate hypercoagulability and some patients may show venous and arterial thromboses, particularly those with severe or critical conditions. All the recommendations suggest patient specific risk stratification for thrombotic events versus haemorrhagic events. Prolonged anticoagulation prophylaxis can be considered for patients with a low risk of bleeding and elevated risk for VTE (venous thromboembolism) (4).

### 3.15 Cognition and mental health

Neurological sequelae of Long COVID can include continuous symptoms such as headache, myalgia, anosmia, dysgeusia, sleep disturbance, difficulty in concentration, PTSD, and depression long after the acute phase of COVID-19. A recent meta-analysis demonstrated that among persistent symptoms, neurocognitive symptoms such as headache (27.8%), myalgia (23.14%), anosmia (22.8%), dysgeusia (12.1%), sleep disturbance (63.1%), confusion (32.6%), difficulty to concentrate (22%), and psychiatric symptoms such as PTSD (31%) and feeling depressed (20%) had a higher prevalence (138).

Presently, there is a lack of studies assessing any pharmacological intervention for neurological sequelae of Long COVID. Reports have been published suggesting a potential benefit of some interventions [e.g., flavonoid luteolin (139), cannabis derivatives cannabidiol and cannabivarin (140), and methylene blue (141)]; however, the research on its practical therapeutical application is still in its nascent stage.

Psychological aspects of Long COVID can be managed as part of the recovery process, but not seen as the primary treatment focus (39, 142). Sleep disturbance can be addressed by following relevant guidelines on insomnia, and a range of treatment strategies can be considered (143). Patients with cognitive problems alongside or as a manifestation of Long COVID can be treated by sticking to the relevant guidelines: depression (144), anxiety (145), PTSD (146) and other mental health problems (147).

### 3.16 Olfactory and gustatory dysfunction

Persistent olfactory and gustatory dysfunctions are one of the most common symptoms of Long COVID. Global studies show a prevalence of 12–56% of long-term anosmia at 4 weeks (60, 113, 148–150), 2–25% at 8 weeks (114, 115, 150) and 13–46% at 12 weeks (118, 151), while for dysgeusia, the rates are 9–50% (60, 113, 148, 152), 1–10% (114, 115), and 11–31% (118, 151), respectively.

The non-neuronal role of the angiotensin-converting enzyme 2 (ACE2) receptor may enable the entry of the SARS-CoV-2 virus into olfactory support cells, stem cells and perivascular cells. This local infection could cause inflammatory response, which may eventually diminish the function of olfactory sensory neurons (153). Additionally, by destructing the support cells responsible for local water and ionic balance, SARS-CoV-2 may indirectly decrease signaling from sensory neurons to the brain, resulting in olfactory dysfunction (153).

In most cases, olfactory and gustatory dysfunction resolve slowly over several weeks and do not require intervention except for education regarding food and home safety. The olfactory training and various topical and systemic treatments were assessed for their effectiveness in managing olfactory dysfunction. Olfactory training demonstrated a benefit of the intervention (154). However, pharmacologic therapy with steroids (nasal or systemic), theophylline, sodium citrate, N-methyl D-aspartate antagonist (caroverine), traditional Chinese acupuncture, a-lipoic acid, vitamin A, minocycline, and zinc sulphate still need to be evaluated for their beneficial effect on olfactory dysfunction (154).

### 3.17 Musculoskeletal symptoms

Acute SARS-CoV-2 infections can cause arthralgia and myalgia in patients. Arthralgia is defined as non-arthritic pain in ≥1 joints without the presence of inflammation (edema, joint pain or heat). Arthralgia may persist in 10–15% of patients at 4 weeks (60, 120) and 16–27% at 8 weeks (115, 120). Muscle pain or myalgia may affect ≥1 muscles and is generally benign and self-limiting. Ligaments, tendons, and fasciae may also be associated. It may persist in 15% of patients at 4 weeks (113), in 6–13% at 8 weeks (114, 115) and in 16% at 12 weeks (118). The management strategy for musculoskeletal symptoms includes symptomatic treatment.

### 3.18 Other commonly reported manifestations

Long COVID may cause organ damage with low or high risk for severe acute disease (155). Studies found that people who recovered from Long COVID exhibited acute kidney injury (156, 157). A study evaluating kidney functionality in patients with COVID-19 found that 35% had decreased kidney function at 6 months post-discharge (158). Long COVID may also impact pancreas, resulting in pancreatitis (159). A cross sectional study found that 40% of patients with mild COVID-19, after 141 days of post COVID infection, showed mild abnormalities of the pancreas (159, 160). Other organs, namely the liver, gastrointestinal tract, and blood vessels express the ACE2 receptor and are susceptible to direct damage from SARS-CoV-2 and indirect damage (161, 162). A gamut of long-term symptoms including general symptoms (fever, chills, intolerance to temperature changes), otolaryngologic symptoms (rhinitis, nasal congestion, tinnitus, vertigo, pain, oropharyngeal discomfort), dryness or conjunctivitis is reported (163). As with other severe major illnesses, COVID-19 can also lead to temporary heavy hair loss weeks after acute disease. Presently, there is a paucity data explaining the mechanisms to most of these symptoms. Relevant symptomatic treatment can be employed for these symptoms (160).

### 3.19 Effect of acute COVID-19 treatment on reducing risk of Long COVID

Although there are limited data, treatment received during acute phase of COVID-19 may have an impact on Long COVID. A recent study showed that treatment with the oral nirmatrelvir within 5 days of a positive SARS-CoV-2 test was associated with reduced risk of 10 of 12 post-acute sequelae of SARS-CoV-2 (HR 0.74, 95% CI: 0.69, 0.81). The effect was consistent regardless of vaccination status or re-infection. The reduced post-acute sequelae included dysrhythmia, ischemic heart disease, deep vein thrombosis, pulmonary embolism, fatigue, liver disease, acute kidney disease, muscle pain, neurocognitive impairment, and shortness of breath (164).

In a study from Italy, treatment with remdesivir demonstrated a 35% reduction in Long COVID symptoms at 6 months follow-up among hospitalized COVID-19 patients (*n =* 449) (165). This study raises a possibility of reducing the risk of Long COVID with other antiviral agents too (166). Recent times, melatonin has been has been studied as a potential early drug treatment for acute COVID-19; it could also have a role in management of Long COVID patients who experience neuropsychiatric symptoms such as insomnia, depression and anxiety (167). Prophylaxis with monoclonal antibodies could reduce the incidence of Long COVID (168). Earlier during the pandemic, the US FDA has authorized concomitant application of tixagevimab plus cilgavimab, for pre-exposure prophylaxis of COVID-19 in patients who cannot be vaccinated against COVID-19 due to allergy or immune compromise (169). A couple of other pairs of antibodies, bamlanivimab plus etesevimab, and casirivimab plus imdevimab were authorized also for post-exposure prophylaxis of COVID-19 (169). However, the effectiveness of monoclonal antibodies (mAbs) has been reduced against the BA.4 and BA.5 Omicron subvariants due to their evolving mutations. Systemic steroids could be helpful in expediting the recovery of Long COVID patients, however more evidence is still required (170, 171). Further, vitamin D due to its immunomodulatory effects is anticipated to be effective in treating long COVID (172, 173). However, there is paucity of data on the association between vitamin D and Long COVID (172, 173). Therefore, more evidence is required on the role of vitamin D on Long COVID.

### 3.20 Proactive measures to reduce the impact of Long COVID

Proactive approaches to prevent Long COVID and spreading awareness will be very helpful in curtailing a part of Long COVID burden. Some of the proactive measures are discussed below.

### 3.21 Vaccination

The impact of vaccination on long COVID has been varied across studies. While some studies report no significant effect, others suggest a reduced risk, with the magnitude of this reduction differing based on factors such as study population, methodology, definition of Long COVID, SARS CoV-2 variant, time since vaccination, double versus triple-vaccination and reinfection rates (20, 57, 174–176).

The mechanisms by which vaccination may reduce the risk of long COVID are yet not fully understood. Proposed mechanisms include reducing the number of SARS-CoV-2 infections, reducing the severity of acute illness and viral load, and consequently minimizing virus-induced pathology and the persistence of viral infections (57). These findings highlight the need for further research to clarify the specific role of vaccination in mitigating long COVID risk and to determine which population may benefit the most.

Considering the implications of Long COVID, it is important to take preventive measures besides avoiding infection. Due to the unavailability of a specific treatment, COVID-19 vaccination plays an important role in reducing severity and transmission, which eventually can reduce the incidence of Long COVID.

Several vaccines have been developed for SARS-CoV-2 since the attack of COVID-19 pandemic, they notably curtail morbidity and fatality from the infection. A recent systematic review analysed that the vaccinated group had a 29% lesser risk for Long COVID versus the unvaccinated group (RR = 0.71, 95% CI: 0.58, 0.87, *p* < 0.01) (177). Further, vaccination showed to be protective effect in those patients who were vaccinated with two doses (RR = 0.83, 95% CI: 0.74, 0.94, *p* < 0.01), but not with one dose (RR = 0.83, 95% CI: 0.65, 1.07, *p* = 0.14) (177). Furthermore, vaccination was effective against Long COVID in patients either vaccinated before SARS-CoV-2 infection (RR = 0.82, 95% CI: 0.74, 0.91, *p* < 0.01) or vaccinated after SARS-CoV-2 infection (RR = 0.83, 95% CI: 0.74, 0.92, *p* < 0.01) (177). Vaccination reduced the risk of cognitive dysfunction/symptoms, kidney diseases/problems, myalgia, and insomnia (177). Another meta-analysis also witnessed similar findings; vaccination demonstrated diminished risks or odds of Long COVID, and two doses were more effective than one dose (178). In the Saudi Arabia there were four vaccines approved for use against SARS-CoV-2: AZD1222 (AstraZeneca), Comirnaty (Pfizer-BioNTech), Spikevax (Moderna) and Jcovden (Johnson & Johnson) (179, 180). Though the impact of COVID-19 vaccination is yet to be studied against Long COVID, data from the current studies from Saudi Arabia show that vaccination against SARS-COV-2 may limit disease severity, length of hospital stay and need for hospitalization (181, 182).

Other studies have investigated the potential association between influenza and Bacillus Calmette–Guérin (BCG) vaccination with COVID-19 infection and disease severity; however, the available data are currently insufficient to draw definitive conclusions (183–185).

The expert panel opined that COVID-19 vaccination is a priority, both to reduce the burden of COVID-19 infection and the Long COVID consequences. Moreover, Long COVID patient visit is an opportunity to update the required vaccines for paediatric, adolescents, and adult populations. Influenza and pneumococcal vaccinations are of special importance particularly for patients with lung affection and/or chronic co-morbidities.

### 3.22 Establishing one stop clinics for Long COVID

The expert panel mentioned that it is essential to establish one-stop or multidisciplinary team (MDT) clinics, wherever possible, to effectively curtail the burden of Long COVID. Typically, an ideal one-stop clinic would consist of a core team and partnership with multi-specialists. The core team can be made up of a pulmonary medicine, physical medicine and rehabilitation and psychology/mental health. The core one-stop clinic needs to have access to various subspecialties (psychiatry, neurology, cardiology, hematology, infectious diseases, nephrology, dermatology, and otolaryngology) for effective management and timely referral of patients with Long COVID.

Furthermore, it is important to integrate primary care clinics with MDT clinics, address issue of loss of follow-up of patients who are on treatment for COVID-19, and explicitly use telemedicine for addressing the limited availability of some subspecialty clinics. In Saudi Arabia, there was a spur in the usage of the 937-call center during the COVID-19 pandemic time, referring that it is a practical strategy for fighting the COVID-19 pandemic (186).

The expert panel opined that one-stop clinic can be established at a tertiary care center, like in the UK and the US. Through one-stop clinics, communication among various subspecialty physicians can be facilitated. Mutual understanding and better team management is important for effective management of symptoms and care through one-stop clinics for Long COVID. Having the access to a senior psychologist in a one-stop clinic can be very helpful in assessing mental health condition and managing mild psychological symptoms. The panelists also opined that establishment of one-stop clinics at all the locations may not be feasible and economically challenging; therefore, the role of primary care and family physicians is important in the initial assessment of the suspected cases and patients during the post-acute phase. During March 2022, the Saudi MOH launched a clinic for the post-COVID-19 condition and patients can book an appointment at the clinic by calling the number (937) (187). A recent study from Saudi Arabia, found that one-stop clinics can expedite patients recruitment, reduce the period between first visit and booking in the operating room (188).

### 3.23 Nutrition and lifestyle medicine

Nutrition has a vital place in the management of chronic illnesses, and proper nutrition can mitigate the manifestations of viral infections as well (189–191). Variation in the incidence of SARS-CoV-2 worldwide demonstrate the probability of nutrition-related epigenetic modifications (192). For example, a meta-analysis demonstrated a clear association of vitamin D insufficiency and increased risk of SARS-CoV-2 infection (OR = 1.43, 95% CI: 1.00, 2.05) (124). Numerous studies have demonstrated the positive effect of vitamin D supplementation in preventing respiratory infections (193, 194). Data from the current studies demonstrate the beneficial role of probiotics in lung and cognitive health (195). Recently, *Lactobacillus plantarum* is found to exhibit antiviral effects in SARS-CoV-2 infected intestinal epithelial cells (196). The role of probiotic supplementation in COVID-19 is yet to be studied. Evidence is accumulating on the impact of lifestyle medicine in improving health and disease prevention. Following the COVID-19 pandemic, lifestyle medicine plays a significant role in managing several Long COVID-related manifestations through better nutrition, physical activity, stress management, and social connection.

### 3.24 Improving disease awareness

Presently, there is a lot of useful, yet conflicting information on the importance of awareness of long COVID in reducing the disease burden is available, emphasizing the need to enhance the awareness. The panel suggested some lectures and awareness campaigns on the Long-COVID symptoms for under-grad/post-grad medical students and primary health care professionals to improve the awareness among medical community. Patient/parent/guardian awareness is also important to effectively manage Long-COVID patients and impart quality and effective treatments. Publishing recommendations is an essential step to impart disease awareness among healthcare professionals. Inter-office distribution through posters, pamphlets etc. and utilizing various social media platforms are some effective modalities to disseminate Long COVID-related information among Long-COVID patients and care takers.

Management protocols for long COVID should also be emphasized in the broader context of managing other viral illnesses. Although the severity of post-COVID syndrome has been observed to be higher than that of other post-viral conditions - likely due to higher immune response - post-viral sequelae from other infections have been underreported and inadequately managed. Addressing these gaps is essential for improving patient outcomes across various viral illnesses.

## 4 Conclusion

Long COVID implications are of increasing concern as SARS-CoV-2 is evolving fast, and numerous variants have expanded abilities to infect patients or evade the protection by offered by vaccination. One of the unanswered and concerning questions is the time to full recovery. Globally, it has been acknowledged that human population need to co-exist with the virus for several years from now; thus, Long COVID will continue to be an international challenge to health care system and economy. Raising public awareness of Long COVID’s risk factors and appropriate management alternatives is crucial for reducing the disease burden. Additionally, future research should focus on exploring pathophysiology of the Long COVID syndrome and specific interventions for early detection and management.

## Data Availability

The original contributions presented in the study are included in the article/supplementary material, further inquiries can be directed to the corresponding author/s.
